# Photo-thermal activation of persulfate for the efficient degradation of synthetic and real industrial wastewaters: System optimization and cost estimation

**DOI:** 10.1007/s11356-024-32728-w

**Published:** 2024-03-04

**Authors:** Hany Abd El-monem, Hani Mahanna, Mohamed El-Halwany, Mahmoud Samy

**Affiliations:** 1 Environmental Engineering, Management and Technology, Faculty of Engineering, Mansoura University, Mansoura, 35516 Egypt; 2 Public Works Engineering Department, Faculty of Engineering, Mansoura University, Mansoura, 35516 Egypt; 3 Engineering Mathematics and Physics Department, Faculty of Engineering, Mansoura University, Mansoura, 35516 Egypt

**Keywords:** Activated persulfate system, Degradation, Dyes, Real wastewater, Reactive radicals

## Abstract

The photo-thermal activation of persulfate (PS) was carried out to degrade various pollutants such as reactive blue-222 (RB-222) dye, sulfamethazine, and atrazine. Optimizing the operating parameters showed that using 0.90 g/L of PS at pH 7, temperature of 90 °C, initial dye concentration of 21.60 mg/L, and reaction time of 120 min could attain a removal efficiency of 99.30%. The degradation mechanism was explored indicating that hydroxyl and sulfate radicals were the prevailing reactive species. The degradation percentages of 10 mg/L of sulfamethazine and atrazine were 83.30% and 70.60%, respectively, whereas the mineralization ratio was 63.50% in the case of real textile wastewater under the optimal conditions at a reaction time of 120 min. The treatment cost per 1 m^3^ of real wastewater was appraised to be 1.13 $/m^3^ which assured the inexpensiveness of the proposed treatment system. This study presents an effective and low-cost treatment system that can be implemented on an industrial scale.

**Supplementary Information:**

The online version contains supplementary material available at 10.1007/s11356-024-32728-w.

## Introduction

Toxic compounds such as dyes, pesticides, and antibiotics can be detected in the aquatic life as a result of the release of industrial effluents to water streams without efficient purification (Al-Mamun et al. 2019; Samy et al. 2021b, 2023; Younes et al. 2021). The aforementioned pollutants harm the aquatic creatures and humans (El-Bendary et al. 2021; Yue et al. 2021). The conventional treatment techniques such as adsorption, electrocoagulation, membrane filtration, and activated sludge cannot attain suitable purification of emerging pollutants (e.g., dyes, pesticides, phenols, antibiotics) due to their toxicity and bio-disobedient (Balarak et al. 2018; Liu et al. 2021; Mahanna and El-Bendary 2022). Further, these treatment techniques suffer from their expensiveness. Additionally, electrocoagulation and activated sludge processes can produce large volumes of sludge (secondary pollutants) which require further treatment (Samy et al. 2020). Therefore, proper remediation of emerging pollutants is paramount to control the hazards associated with their release to water streams.

Recently, advanced oxidation technologies (AOTs) such as photocatalysis, Fenton, and ozonation have been employed for remediating emerging contaminants effectively (Ghorbani and Salem 2021; Mensah et al. 2022; Zheng et al. 2022; Mahanna et al. 2024). However, the large-scale application of these techniques is still constrained due to the high-cost of photocatalysts and ozone and the production of sludge and the need for low pH values in the case of Fenton (Kakavandi et al. 2019; Zhang et al. 2019; Li et al. 2022; Fawzy et al. 2022). Among AOTs, sulfate radicals and sulfite-based AOTs have occupied remarkable position due to the effective degradation of emerging pollutants as a result of the generation of sulfate radicals with higher oxidation potential and longer half-life time compared to hydroxyl radicals that are mainly generated in other AOTs (Reza Samarghandi et al. 2020; Wu et al. 2023). Further, other reactive substances such as hydroxyl radicals and superoxide radicals can be generated in sulfate radicals and sulfite-based AOTs which contribute to the enhancement of the degradation performance (Wu et al. 2021; Rahmani et al. 2022). Moreover, non-radical species such as singlet oxygen can be generated in sulfate radicals and sulfite-based AOTs. The reactive radicals have higher oxidation potential than the non-radical species (Zhang et al. 2024). However, the non-radical species have better selectivity towards the pollutants (Zhang et al. 2024). Persulfates such as peroxymonosulfate (PMS, HSO_5_^−^) and peroxydisulfate (Persulfate (PS), S_2_O_8_^−^) and sulfite (SO_3_^2−^) can act as precursors of sulfate radicals in the case of their activation (Wang et al. 2024). In this study, the employment of persulfate as a precursor of sulfate radicals was performed. The oxidation potential of persulfate ions (PS, S_2_O_8_^2−^) is 2.01 V, while the oxidation power of sulfate radicals (SO_4_^•−^) that generate as a result of the activation of S_2_O_8_^2−^ is 2.6 V. Thus, activating PS to generate more powerful radicals can improve the degradation performance. PS can be activated via the cleavage of peroxide bond using heat, ultrasound, and UV. Further, it can be activated via a redox reaction using transition metals and carbonaceous materials (Li et al. 2020). The use of transition metals as activators for PS can lead to the metal ions leaching into the aqueous solutions which requires post treatment (Feng et al. 2022). The generated sulfate radicals can oxidize the pollutants to simpler by-products and harmless compounds such as CO_2_ and H_2_O.

In this study, the activation of PS was performed using combined effects of heat and UV for the remediation of reactive blue-222 dye, sulfamethazine (antibiotic), and atrazine (pesticide). The effects of operating conditions such as initial pollutant concentration, persulfate concentration, pH, and temperature were explored. Further, the degradation mechanism was investigated, and the effects of organic matter, turbidity, and inorganic salts in real textile wastewater on the degradation performance were investigated. Additionally, the degradation of other pollutants such as sulfamethazine and atrazine was performed using the proposed system.

## Materials and methods

### Materials

Sodium persulfate (Na_2_S_2_O_8_, 99.9%), sodium azide (NaN_3_, 99%), tert-butyl alcohol (C_4_H_10_O, 97%), benzoquinone (C_6_H_4_O_2_, 99%), zinc chloride (ZnCl_2_, 95%), copper chloride dihydrate (CuCl_2_.2H_2_O, 97%), sodium sulfate (Na_2_SO_4_, 98%), sodium phosphate dodecahydrate (Na_3_PO_4_.12H_2_O, 95%), and ethanol (C_2_H_6_O, 99%) were purchased from Sigma Aldrich. Sulfamethazine (C_12_H_14_N_4_O_2_S, 99%) and atrazine (C_8_H_14_ClN_5_, 99.5%) were procured from Alfa Aesar. Reactive blue-222 dye (C_37_H_24_N_10_Na_6_O_22_S_7_, 99%) was obtained from Oh Young Industrial Co. Ltd., South Korea.

### Real textile wastewater

Real textile wastewater was compiled from the industrial zone in Damietta, Egypt. Then, the collected samples were saved at room temperature and left for 24 h to allow the settling of the suspended solids. Subsequently, 500 mL of the sample was decanted for experimentation. The properties of real textile wastewater is provided in Table 1.

**Table 1:** Characteristics of real textile wastewater

Parameter	Unit	Value
pH	-	7.7
Total dissolved solids (TDS)	mg/L	600
Conductivity	mS/cm	0.091
Turbidity	NTU	3.3
Total organic carbon (TOC)	mg/L	123.5

### Experimental procedures

The activation of persulfate was performed in a 250 mL Pyrex beaker filled with 100 mL of the contaminated solution as shown in Fig. (S1). A magnetic stirrer with hot plate was used to mix the solution and control the temperature and the temperature was measured periodically using a thermometer. Further, metal halide lamp (400 W, Venture) was used as a light source. The spectrum of metal halide lamp was provided in the supplementary file (Fig. S2). The degradation of reactive blue-222 dye was initially attained by raising the temperature to 50 °C without adding persulfate (PS) salts and without the lamp. Moreover, the degradation of the dye was performed using the lamp without adding PS at a room temperature (30 °C). Additionally, the removal of the dye was carried out using PS only without the lamp at a room temperature (30 °C), PS with the lamp, PS with heat (50 °C), and PS with the lamp and heat (50 °C). The effect of pH (3–11) was also explored.

The optimization and modeling of the operating parameters such as PS concentration, temperature, and dye concentration were performed by conducting 15 experiments according to response surface methodology (RSM) and central composite design (CCD) as shown in Table 2 and 3. The strength of the obtained model was evaluated by analysis of variance (ANOVA). On the other hand, the optimum conditions were also used to perform the degradation of sulfamethazine (10 mg/L) and atrazine (10 mg/L).

**Table 2:** Optimization of operating parameters

Independent variables	Units	Levels				
		-2	-1	0	1	2
Initial dye concentration	mg/L	15	20	25	30	35
PS concentration	g/L	0.1	0.3	0.5	0.7	0.9
Temperature	°C	10	30	50	70	90

**Table 3:** Degradation efficiencies of reactive blue-222 dye under different operating conditions

Coded values				Real values		Removal percentage of the dye		
Run	Dye concentration	PS concentration	Temperature	Dye concentration	PS concentration	Temperature	Lab-work	Expected
1	1	1	1	30	0.7	70	57.22	53.616
2	1	-1	1	30	0.3	70	31	35.252
3	1	1	-1	30	0.7	30	40.45	38.772
4	1	-1	-1	30	0.3	30	16.86	15.816
5	-1	1	1	20	0.7	70	76.6	80.586
6	-1	-1	1	20	0.3	70	48	52.622
7	-1	1	-1	20	0.7	30	61.8	60.502
8	-1	-1	-1	20	0.3	30	21.4	27.946
9	-2	0	0	15	0.5	50	77.9	72.36
10	2	0	0	35	0.5	50	30.8	33.26
11	0	0	2	25	0.5	90	85.7	82.454
12	0	0	-2	25	0.5	10	42.77	42.934
13	0	2	0	25	0.9	50	48.1	50.762
14	0	-2	0	25	0.1	50	5.6	3.7
15	0	0	0	25	0.5	50	44.8	41.75

The main reactive species were specified using ethanol as a quencher of sulfate radicals and hydroxyl radicals, tert-butyl alcohol as a scavenger of hydroxyl radicals, sodium azide as a scavenger of singlet oxygen and benzoquinone as a scavenger of superoxide radicals. The concentration of scavenger is 50 mM. The effects of inorganic ions such as cations (Zn^2+^ and Cu^2+^) and anions (SO_4_^2−^ and PO_4_^3−^) with a concentration of 10 mg/L were explored.

### Analytical methods

The concentrations of sulfamethazine and atrazine were measured as reported in our previous studies (Samy et al. 2020; El-Bestawy et al. 2023). Further, the concentration of reactive blue-222 (RB-222) dye was measured using a UV–Vis spectrophotometer (Shimadzu) at a wavelength of 605 nm. Additionally, the intermediates generated during the degradation of RB-222 were determined using liquid chromatography-mass spectroscopy (LC–MS, Shimadzu) using the method described by Kore et al. (2023) (Kore et al. 2023).

Regarding the real textile wastewater, total organic carbon was quantified using total organic carbon analyzer (TOC, Shimadzu). Also, pH, total dissolved solids, and conductivity were measured by a multiparameter analyzer (Extech 341350A Oyster Series), and the turbidity was estimated by a turbidimeter (Turb 430 T, WTW, USA).

## Results and discussion

### Degradation of RB-222 dye using photo-thermal activated persulfate system

**Fig. 1:**
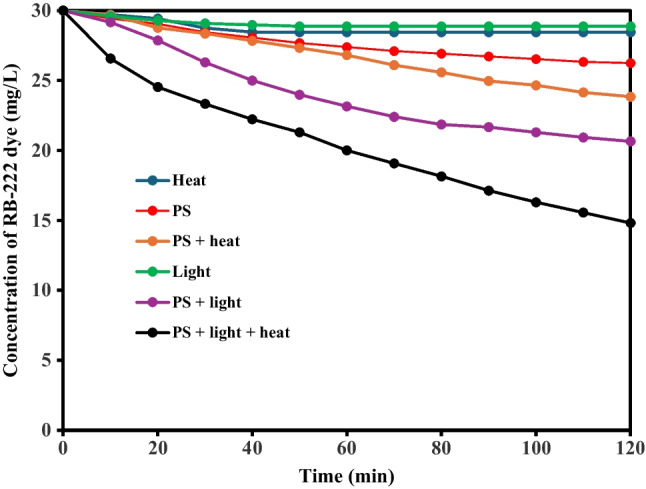
Degradation of RB-222 dye using heat/light activated persulfate system

The degradation of RB-222 was attained using single-PS, single-light, single-heat, PS with heat, PS with light, and PS with light and heat as shown in (Fig. 1) at pH 7, initial dye concentration of 30 mg/L, PS concentration of 0.50 g/L, reaction time of 120 min, and temperature of 50 °C. The degradation percentages of RB-222 using single-heat and single-light were only 5.20% and 3.76%, respectively due to the complexity of the dye (Wang and Wang 2018). Further, the degradation of RB-222 by single-PS was not high (12.60%) due to the low oxidation potential of persulfate ions (2.01 V) (Đurkić et al. 2021). On the other hand, the activation of PS by the light or heat improved the degradation ratios to 31.33% and 20.67% due to the generated radicals such as sulfate radicals, hydroxyl radicals, superoxide radicals, and singlet oxygen as a result of the activation of PS by the heat or light (He et al. 2021). Further, the photo-thermal activation of PS raised the degradation ratio to 50.70% due to the increase of the generated reactive species owing to the synergetic effects of heat and light on the activation of PS. Thus, in the next experiments, light/heat-activated persulfate system was employed.

### Effect of pH on the degradation performance

**Fig. 2:**
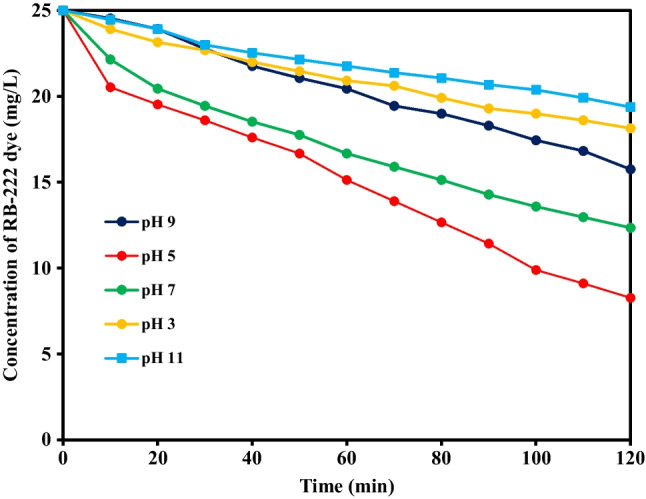
Degradation of RB-222 dye under different pH values

The degradation efficiencies of RB-222 dye were explored at different pH values (3–11), initial dye concentration of 25 mg/L, PS concentration of 0.50 g/L, reaction time of 120 min, and temperature of 50 °C as depicted in (Fig. 2). The degradation ratios of RB-222 dye were 28%, 67%, 50.80%, 37.20%, and 22.40% at pH values of 3, 5, 7, 9, and 11, respectively. At alkaline conditions, the major reactive species would be hydroxyl radicals that own shorter half-life time and lower oxidation ability compared to sulfate radicals as shown in Eq. (1) which could decrease the degradation efficiency (Liu et al. 2016). Further, the generated hydroxyl radicals could scavenge sulfate radicals as given in Eq. (2) (Norzaee et al. 2017). Additionally, the abundant hydroxyl ions at pH above 7 might act as deactivators for the generated hydroxyl radicals (Eq. (3)) (Waldemer et al. 2007). On the other hand, at pH 5 and 7, the degradation efficiencies were higher due to the prevalence of sulfate radicals with longer lifetime and higher oxidation potential which improved the degradation of the dye (Huang et al. 2002). Further, at low pH values, the decomposition of PS was higher compared to alkaline conditions which resulted in the availability of abundant persulfate ions, thereby improving the degradation performance. Howbeit, decreasing the pH to extreme values (pH 3) could inhibit the accelerated degradation of RB-222 due to the quenching nature of sulfate radicals as shown in Eq. (4) (Huling et al. 2011). Norzaee et al. (2017) reported that the highest degradation of penicillin was attained at pH 5 in a UV-activated PS system (Norzaee et al. 2017). Next experiments were performed under various operating parameters to maximize the degradation efficiency of RB-222 dye at pH 7.

1 $${{{\text{SO}}}_{4}}^{\bullet -}+{{\text{OH}}}^{-}\to {{\text{OH}}}^{\bullet }+{{{\text{SO}}}_{4}}^{2-}\left(\mathrm{Alkaline\;pH}\right)$$

2 $${{\text{OH}}}^{\bullet }+{{{\text{SO}}}_{4}}^{\bullet -}\to {{{\text{HSO}}}_{4}}^{-}+{0.5{\text{O}}}_{2}$$

3 $${{\text{OH}}}^{\bullet }+{{\text{OH}}}^{-}\to {{\text{O}}}^{-}+{{\text{H}}}_{2}{\text{O}}$$

4 $${{{\text{SO}}}_{4}}^{\bullet -}+{{{\text{SO}}}_{4}}^{\bullet -}\to {{\text{S}}}_{2}{{{\text{O}}}_{8}}^{2-}$$

### Optimal operating parameters

A polynomial model as given in Eq. (5) was obtained to connect the degradation percentage of RB-222 dye with the operating parameters such as initial dye concentration, temperature, and PS concentration as shown in Table 3. The suitability of the model (R^2^ = 97.15%) was affirmed through the P and F values as explained in Table 4.

5 $${\text{R}}\left(\mathrm{\%}\right)=56.7-5.63\mathrm{ X}+240.8\mathrm{ Y}-0.344\mathrm{ Z}+0.1106{\mathrm{ X}}^{2}-102.8 {{\text{Y}}}^{2}+0.01309{\mathrm{ Z}}^{2}-2.40\mathrm{ XY}-0.0131\mathrm{ XZ}-0.287\mathrm{ YZ}$$

where R (%) is the removal ratio of RB-222 dye, X, Y, and Z are initial dye concentration (mg/L), PS concentration (g/L), and temperature (°C), respectively.

**Table 4:** ANOVA for the degradation efficacy of the reactive dye

Source	DF	Sum of squares	Mean square	F-value	P-value
Model	9	7285.52	809.5	18.95	0.002
Linear	3	5689.95	1896.65	44.4	0.001
RB-222 concentration (mg/L)	1	1530.18	1530.18	35.82	0.002
PS concentration (g/L)	1	2596.16	2596.16	60.77	0.001
Temperature (°C)	1	1563.61	1563.61	36.6	0.002
Square	3	1525.27	508.42	11.9	0.01
RB-222 concentration (mg/L) * RB-222 concentration (mg/L)	1	84.65	84.65	1.98	0.218
PS concentration (g/L) * PS concentration (g/L)	1	187.16	187.16	4.38	0.091
Temperature (°C) * Temperature (°C)	1	303.65	303.65	7.11	0.045
2-Way Interaction	3	70.3	23.43	0.55	0.671
RB-222 concentration (mg/L) * PS concentration (g/L)	1	46.03	46.03	1.08	0.347
RB-222 concentration (mg/L) * Temperature (°C)	1	13.76	13.76	0.32	0.595
PS concentration (g/L) * Temperature (°C)	1	10.51	10.51	0.25	0.641
Error	5	213.59	42.72		
Total	14	7499.11			

**Fig. 3:**
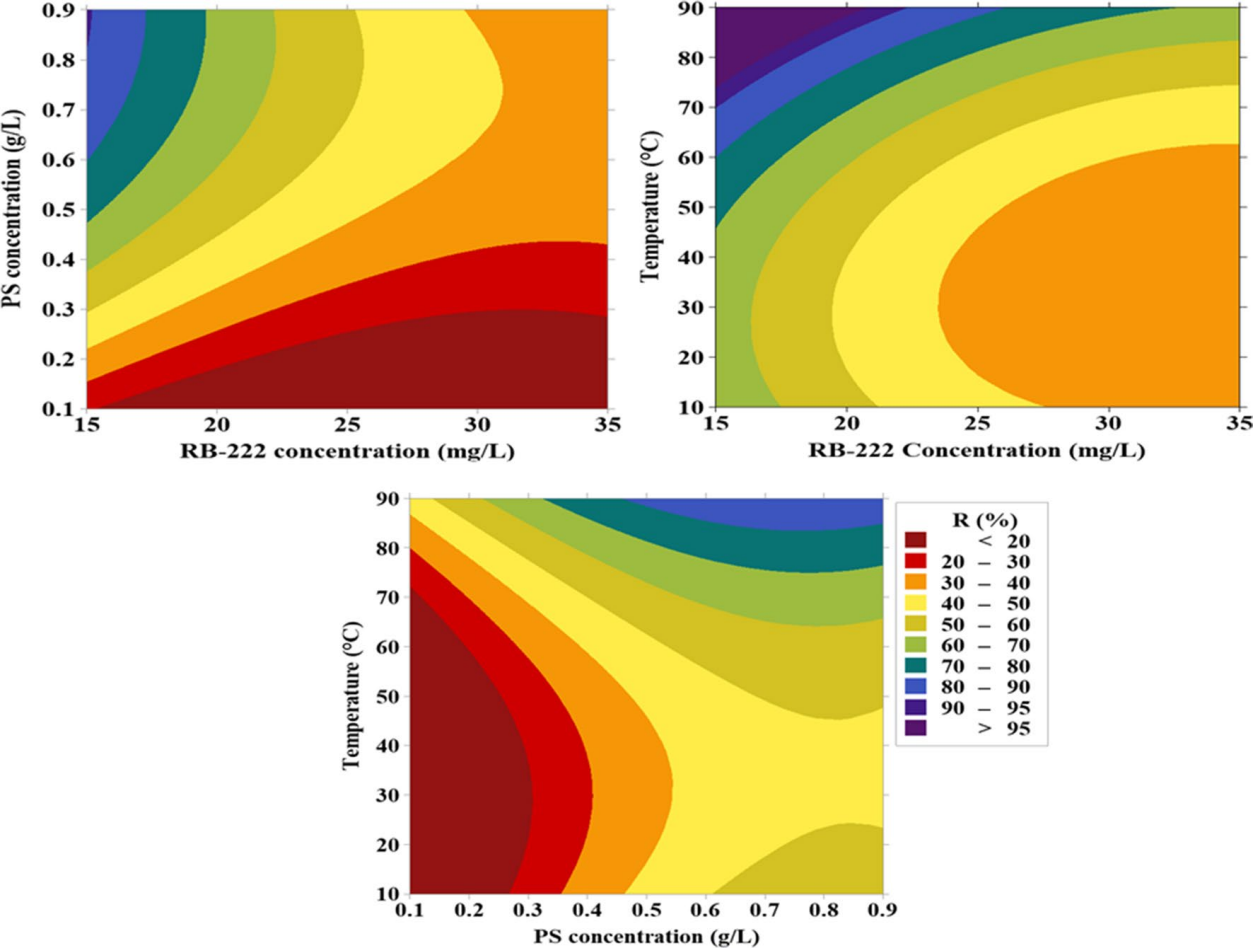
Removal efficiencies of RB-222 dye under different operating parameters

Figure 3 portrays the removal efficiencies of RB-222 under different operating conditions. The results showed that raising the dye concentration above 21.60 mg/L could decrease the degradation performance. At low dye concentrations, the generated radicals are enough to achieve high degradation percentages, whereas the degradation ratios were not high at high concentrations of the dye at the same time due to the need for more radicals which requires the extension of time (Song et al. 2022).

The optimum PS concentration was 0.90 g/L. The increase of PS ions could participate in increasing the generated radicals, thereby raising the degradation efficiency (Liu et al. 2016). However, raising the PS concentration does not always improve the degradation performance due to the quenching effect of PS ions at high concentrations (El-Bestawy et al. 2023).

Regarding the effect of temperature, the increase of temperature could provide more energy to cleave peroxide bond (O–O) in persulfate which would increase the degradation efficiency owing to the raising of the generated reactive species (Gao et al. 2015). The optimum values of initial dye concentration, PS concentration, and temperature are listed in Table 5. The experimental degradation efficiency of RB-222 was 99.30% under the optimal condition compared to 99.90% obtained from the model which assured the model’s applicability.

**Table 5:** Optimum operating parameters

Parameters	Optimum values
Initial dye concentration (mg/L)	21.6
PS concentration (g/L)	0.9
Temperature (°C)	90
Predicted removal ratio of the dye (%) after 120 min under the optimal conditions	99.9
Experimental degradation percentage of the dye (%) after 120 min under the above conditions	99.3

### Degradation mechanism of RB-222 dye in heat/light activated persulfate system

The activation of PS ions by heat and light could result in the rupture of O–O bond in PS and the generation of sulfate radicals as shown in Eq. (6). Then, the generated sulfate radicals could react with H_2_O to generate hydroxyl radicals as given in Eq. (7). Additionally, superoxide radicals could be generated in heat/light-activated PS system via the reaction of PS ions with HO_2_^−^ as described in Eqs. (8,9). Further, singlet oxygen (Non radical pathway) could be formed as given in Eqs. (10,11) as a result of the reaction between superoxide radicals and hydroxyl radicals or/and via the reaction between H^+^ and superoxide radicals. Due to the variety of the generated radicals in this system, the major reactive species were determined as presented in the next sections.

The reactive species could attack the functional groups of the RB-222 dye leading to the generation of 1,8-diaminonaphthalene-2,3,6,7-tetraol, phthalic acid, and 3,4-dihydroxy-benzoic acid (Shokoohi et al. 2023). Then, the reactive radicals would cleave the chains in the aforementioned intermediates resulting in the formation of simpler by-products such as pentanoic acid, propane-1,2,3-triol, 2-oxopropanoic acid, and propionic acid (Shokoohi et al. 2023). The frequent attack of the reactive species on the RB-222 dye and the generated by-products could transform them to harmless compounds such as CO_2_ and H_2_O. The degradation pathways were suggested and provided in the supplementary file (Fig. S3).

6 $${{\text{S}}}_{2}{{{\text{O}}}_{8}}^{2-}\stackrel{{\text{Heat}}/{\text{light}}}{\to }2 {{{\text{SO}}}_{4}}^{\bullet -}$$

7 $${{{\text{SO}}}_{4}}^{\bullet -}+{{\text{H}}}_{2}{\text{O}}\to {{{\text{SO}}}_{4}}^{2-}+{{\text{OH}}}^{\bullet }+{{\text{H}}}^{+}$$

8 $${2{\text{H}}}_{2}{\text{O}}+{{\text{S}}}_{2}{{{\text{O}}}_{8}}^{2-}\to {{{\text{SO}}}_{4}}^{2-}+{3{\text{H}}}^{+}+{{\text{HO}}}_{{2}^{-}}$$

9 $${{\text{HO}}}_{{2}^{-}}+{{\text{S}}}_{2}{{{\text{O}}}_{8}}^{2-}\to {{{\text{SO}}}_{4}}^{2-}+{{\text{H}}}^{+}+{{{\text{O}}}_{2}}^{\bullet -}$$

10 $${{\text{OH}}}^{\bullet }+{{{\text{O}}}_{2}}^{\bullet -}\to {}^{1}{{\text{O}}}_{2}+{{\text{OH}}}^{-}$$

11 $${2{\text{H}}}^{+}+{{2{\text{O}}}_{2}}^{\bullet -}\to {}^{1}{{\text{O}}}_{2}+{{\text{H}}}_{2}{{\text{O}}}_{2}$$

**Fig. 4:**
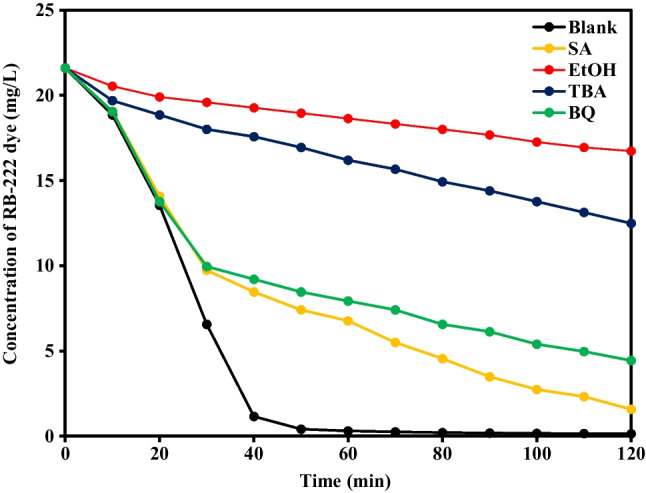
Determination of major reactive species

Different quenchers such as ethanol (EtOH, 50 mM), tert-butyl alcohol (TBA, 50 mM), benzoquinone (BQ, 50 mM), and sodium azide (SA, 50 mM) were used to deactivate (sulfate and hydroxyl radicals), hydroxyl radicals, superoxide radicals, and singlet oxygen at pH 7, initial dye concentration of 21.60 mg/L, PS concentration of 0.90 g/L, and temperature of 90 °C as given in (Fig. 4). The lowest degradation ratios were attained after adding TBA and EtOH, where the degradation percentages were 42.10% and 22.50% in the case of TBA and EtOH, respectively compared to 99.30% in the case of the blank solution (without quencher) which affirmed the main role of hydroxyl and sulfate radicals in the degradation process. On the other hand, the degradation percentages slightly decreased after adding SA and BQ which stated the minor contribution of singlet oxygen and superoxide radicals in the degradation system compared to hydroxyl and sulfate radicals.

### Degradation of other organic pollutants and real industrial effluents

**Fig. 5:**
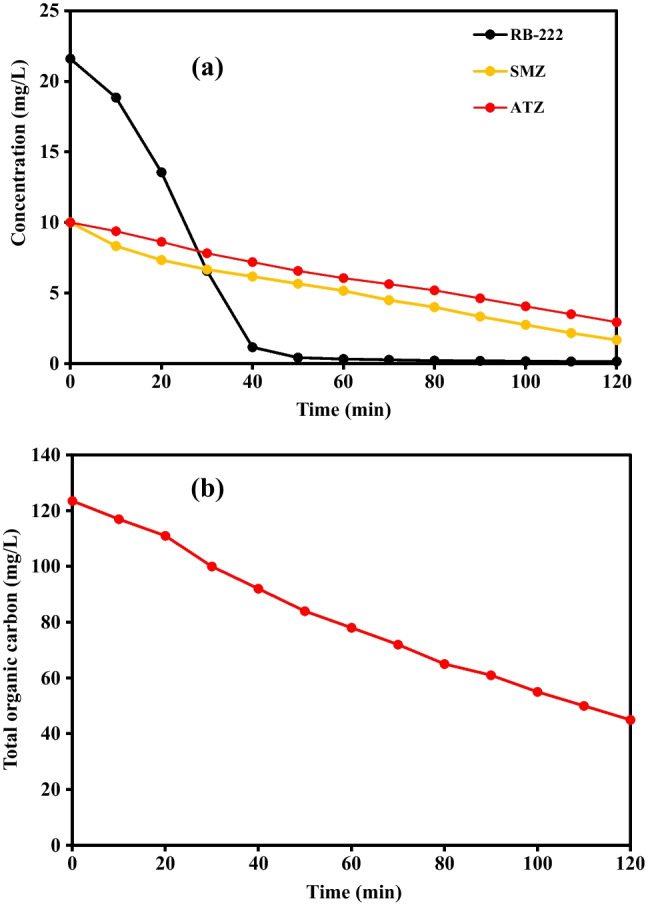
(**a**) Degradation of SMZ and ATZ and (**b**) TOC mineralization of real textile wastewater

Sulfamethazine (SMZ) and atrazine (ATZ) degradation with an initial concentration of 10 mg/L was performed at pH 7, PS concentration of 0.90 g/L, and temperature of 90 °C as given in (Fig. 5a). The removal efficiencies of SMZ and ATZ were 83.30% and 70.60%, respectively affirming the potential of our system to degrade different types of pollutants. Further, the degradation rates of SMZ, ATZ, and RB-222 were estimated using Langmuir–Hinshelwood kinetic model (Fig. S4) as reported in our previous study (Samy et al. 2023). The degradation rates were 0.0514, 0.013, and 0.0091 min^−1^ in the case of RB-222, SMZ, and ATZ, respectively. The degradation ratios and rates in the case of SMZ and ATZ were not high compared to RB-222, as the used operating parameters were the optimum values for the degradation of RB-222. To achieve higher degradation in the case of SMZ and ATZ, an optimization study for each pollutant has to be performed to specify the best operating conditions that can attain the highest degradation in the case of SMZ and ATZ.

The effects of inorganic ions such as cations (Zn^2+^ and Cu^2+^) and anions (SO_4_^2−^ and PO_4_^3−^) were investigated as given in Fig. (S5) at pH 7, initial dye concentration of 21.6 mg/L, PS concentration of 0.9 g/L, and temperature of 90 °C. The degradation ratios declined to 65% and 60% in the case of Zn^2+^ and Cu^2+^, respectively, whereas the degradation percentages decreased to 71% and 77% in the case of SO_4_^2−^ and PO_4_^3−^, respectively compared to 99.3% removal efficiency in the case of the absence of inorganic ions. The decrease of degradation efficiencies in the case of the presence of cations and anions might be due to the ability of inorganic ions to react with the reactive species generating radicals with lower oxidation potential. Further, the inorganic ions could serve as a quencher of the reactive species which resulted in the decrease of degradation performance.

On the other hand, the proposed degradation system was employed for the degradation of real textile wastewater as depicted in (Fig. 5b). The characteristics of the real industrial effluents are listed in Table 1. The real wastewater exhibited high concentrations of dissolved organic carbon and inorganic salts as well as high turbidity. Further, the real wastewater is a complex matrix that contains different types of pollutants. The increase of turbidity might obstruct or scatter the photons that could activate the PS, thereby inhibiting the degradation rate (Luna-Sanguino et al. 2021). Additionally, the organic matter and inorganic salts could scavenge the generated radicals (Metheniti et al. 2018). Moreover, the inorganic ions viz., nitrates, chlorides and sulfates could form reactive radicals with lower oxidation ability as a result of the reaction with the main reactive species (Samy et al. 2021a). The TOC was measured with time to express the degradation performance due to the complexity of real wastewater matrix. The TOC mineralization percentage was only 19% after 30 min due to the the presence of inorganic ions, organic matter, and turbidity that could deaccelrate the degradation rate due to the above-mentioned explanations. Further, the attack of radicals on the organic matter could form organic by-products. Thus, the time has to be prolonged to produce more radicals to degrade the original organics and the organic intermediates. Thus, the extension of time to 120 min increased the TOC removal percentage to 62.50%. Higher mineralization needs longer time to produce enough reactive species.

### Estimation of the treatment cost using the photo-thermal activated persulfate system

Evaluation of the system’s total cost under the optimal conditions is a significant step to apply this technique on a wider scale. The total cost comprises the operating and amortization costs. The amortization cost includes the costs of construction (Reinforced concrete), mixers, pumps, heaters, and lamps. The cost study was performed based on a lifetime (n) of 25 years, 300 d as a number of working days per year (D), cycle’s time (t_c_) of 3 h (2 h reaction time and 1 h for the preparation), and 12 h as working hours per day (t_w_). The costs were determined per one m^3^ of the real wastewater. The real wastewater is assumed to be treated in successive cycles for treating 20 m^3^/day. Text S1 shows the details of cost estimation. The reactor’s volume was estimated using Eq. (1) in the supplementary file and it was 5 m^3^. Further, Eq. (2) in Text S1 was used to estimate the amortization cost (AC) and the annual AC is 0.026 $/m^3^.

The operating costs were quantified taking into consideration the cost of energy consumption, maintenance, and chemicals besides neglecting the labor costs. The cost of chemicals was roughly 0.9 $/m^3^, and the energy cost was approximately 0.2 $/m^3^. The cost of energy was measured using Eq. (4) in Text S1. Additionally, the maintenance cost was taken as 20% of the annual AC. Thus, the total cost was estimated to be 1.13 $/m^3^. The sensible cost of the proposed system strengths its opportunity to be accepted by the decision makers as a large-scale system for degrading real industrial wastewaters.

## Conclusions

The combined effects of light and heat could efficiently activate PS to attain effective degradation of RB-222 dye, sulfamethazine, and atrazine. The degradation of RB-222 dye was conducted under different values of pH, PS dose, dye concentration, and temperature, and the optimum values were determined. The degradation of RB-222 dye was mainly due to the attack by hydroxyl and sulfate radicals. The degradation efficiencies of RB-222 dye, sulfamethazine, and atrazine were 99.30%, 83.30%, and 70.60% at PS concentration of 0.90 g/L, pH 7, temperature of 90 °C, initial dye concentration of 21.60 mg/L, sulfamethazine and atrazine concentration of 10 mg/L, and reaction time of 120 min. Further, the TOC mineralization ratio reached 63.50% in the case of real textile wastewater under the optimal conditions within 120 min. The cost study exhibited the reasonable cost of treating the real textile wastewater using the proposed treatment system. The high degradation performance along with the inexpensiveness of the proposed system can support the recruitment of this system for treating industrial effluents on a larger scale.

### Supplementary Information

Below is the link to the electronic supplementary material.

Supplementary file1 (DOCX 306 KB)

## Data Availability

All data generated or analyzed during this study are included in this published article (and its supplementary information files).
