# Supplementary material for: Photo-thermal activation of persulfate for the efficient degradation of synthetic and real industrial wastewaters: System optimization and cost estimation
Source: Environ Sci Pollut Res Int. 2024 Mar 4;31(16):24153–62. doi: 10.1007/s11356-024-32728-w (PMC11289345; doi:10.1007/s11356-024-32728-w)
Supplement: Supplementary file 1 — Supplementary file1 (DOCX 306 KB) [file 11356_2024_32728_MOESM1_ESM.docx]

**Photo-thermal activation of persulfate for the efficient degradation of synthetic and real industrial wastewaters: System optimization and cost estimation**

Hany Abd El-monem ^a^, Hani Mahanna ^b,^[[1]](#footnote-1)^^, M.M. El-Halwany ^c^, Mahmoud Samy ^b^

^a^ Environmental Engineering, management and technology, Faculty of Engineering, Mansoura University, Mansoura, 35516, Egypt

^b^ Public Works Engineering Department, Faculty of Engineering, Mansoura University, Mansoura, 35516, Egypt

^c^ Engineering Mathematics and Physics Department, Faculty of Engineering, Mansoura University, Mansoura, 35516, Egypt.


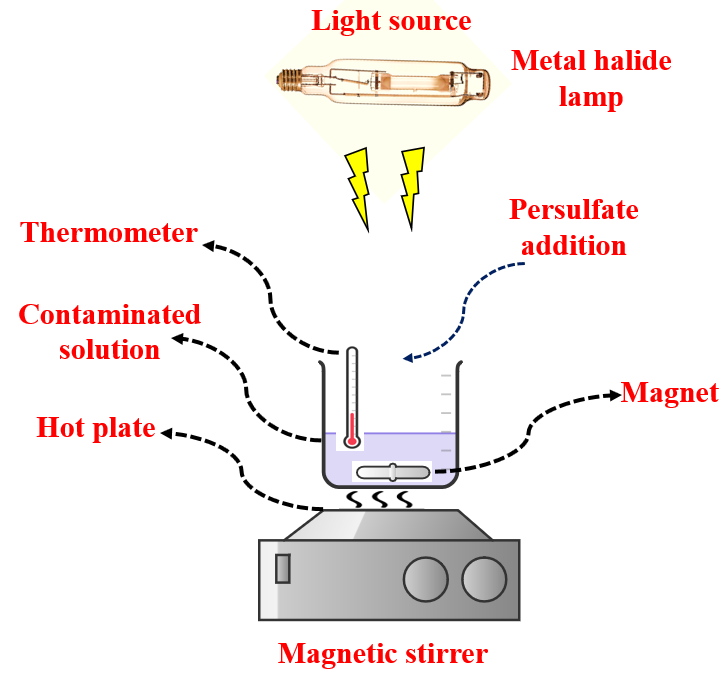


**Fig. S1.** Description of the experimental device.


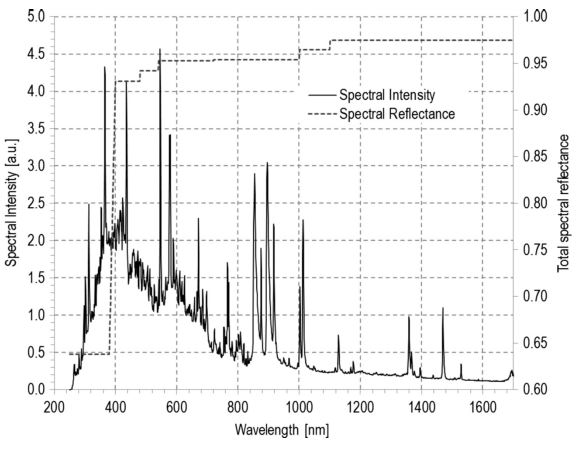


**Fig. S2.** Emission profile for a typical metal halide lamp (Roba and Siegel 2017).


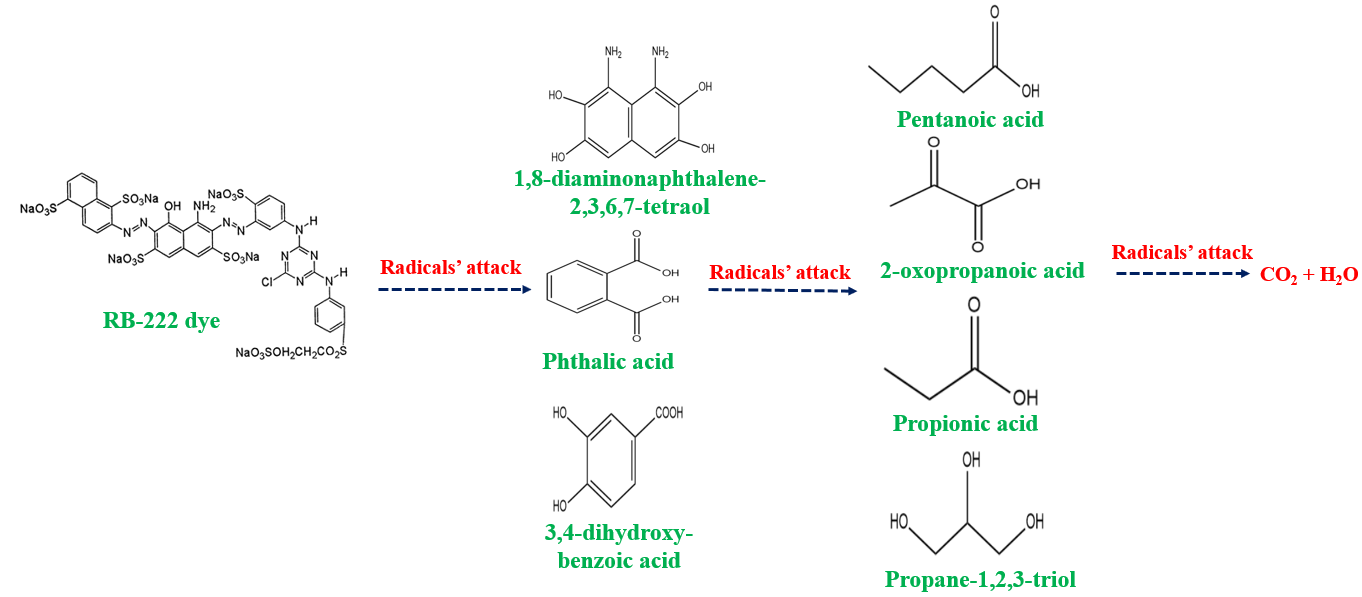


**Fig. S3.** Degradation pathways of RB-222.

**Fig. S4.** Degradation kinetics of the photo-thermal activated persulfate system.


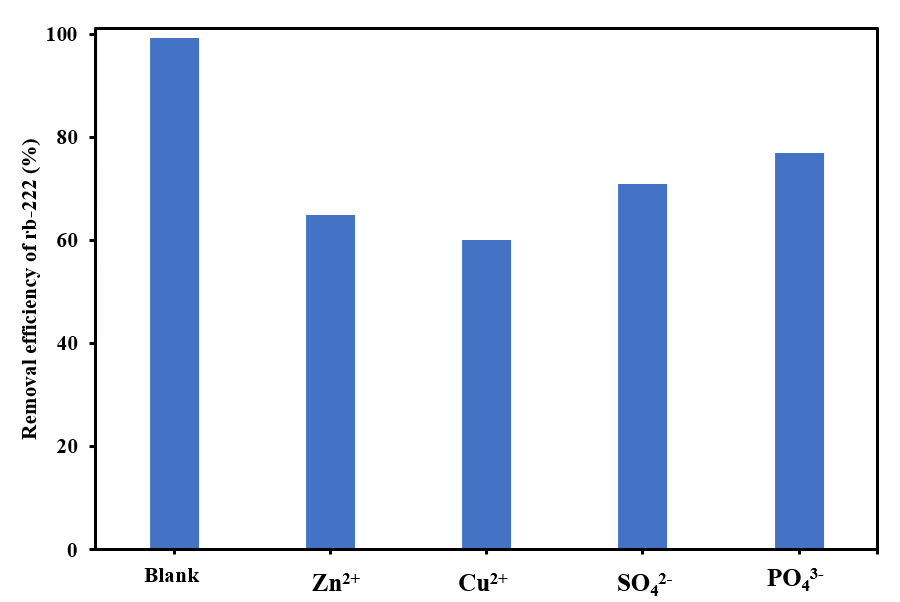


**Fig. S5.** Effects of inorganic ions on the removal efficiency of RB-222.

**Text S1:**

The volume of the reactor (V_r_) was estimated according to Eq. (1) (Gar Alalm et al. 2014):

$$V_{r}=\frac{V_{t}}{D}.\frac{t_{c}}{t_{w}} \left( 1 \right)$$

where V_t_ is the treated volume of wastewater per year (m^3^/year). The AC per m^3^ of the industrial wastewater was estimated as given in Eq. (2) (Gar Alalm and Nasr 2018):

$$AC=\frac{C_{m}}{n}.\frac{V_{r}}{V_{t}} \left( 2 \right)$$

where C_m_ is the cost of constructing the reactor and permanent facilities.

Eq. (3) was used to measure the annual AC:

$${AC}_{annual}=AC (\frac{\left( 1+i \right)^{n} . i}{\left( 1+i \right)^{n}-1}) (3)$$

where i is the interest rate )6%). The V_t_ is assumed to be 6000 m^3^.

The energy consumed for the operation of pumps, heaters, lamps and mixers was estimated in $/m^3^ according to Eq. (4) (Gar Alalm et al. 2015):

$$EC=\frac{{E . t_{w}. D . P}_{E}}{V_{t}} \left( 4 \right)$$

where EC is the energy consumption cost in $/m^3^, E denotes the required power in KW and P_E_ denotes the energy unit price (0.2 $/KW.h).

**References**

Gar Alalm M, Nasr M (2018) Artificial intelligence, regression model, and cost estimation for removal of chlorothalonil pesticide by activated carbon prepared from casuarina charcoal. Sustain Environ Res 28:101–110. https://doi.org/10.1016/j.serj.2018.01.003

Gar Alalm M, Tawfik A, Ookawara S (2014) Solar photocatalytic degradation of phenol by TiO_2_/AC prepared by temperature impregnation method. Desalin Water Treat 1–10. https://doi.org/10.1080/19443994.2014.969319

Gar Alalm M, Tawfik A, Ookawara S (2015) Comparison of solar TiO_2_ photocatalysis and solar photo-Fenton for treatment of pesticides industry wastewater: Operational conditions, kinetics, and costs. J Water Process Eng 8:55–63. https://doi.org/10.1016/j.jwpe.2015.09.007

Roba JP, Siegel NP (2017) The design of metal halide-based high flux solar simulators: Optical model development and empirical validation. Sol Energy 157:818–826. https://doi.org/10.1016/j.solener.2017.08.072

1. Corresponding authors:

   E-mail address: hany_mss@mans.edu.eg [↑](#footnote-ref-1)
